# Involvement of spinal orexin A in the electroacupuncture analgesia in a rat model of post-laparotomy pain

**DOI:** 10.1186/1472-6882-12-225

**Published:** 2012-11-22

**Authors:** Xiao-Ming Feng, Wen-Li Mi, Fang Xia, Qi-Liang Mao-Ying, Jian-Wei Jiang, Sheng Xiao, Zhi-Fu Wang, Yan-Qing Wang, Gen-Cheng Wu

**Affiliations:** 1 Department of Integrative Medicine and Neurobiology, Institute of Acupuncture Research, WHO Collaborating Center for Traditional Medicine, State Key Laboratory of Medical Neurobiology, Institutes of Brain Science, Shanghai Medical College, Fudan University, Shanghai, 20032, China; 2 Shanghai Institute of Acupuncture-Moxibustion and Meridian, Shanghai, 200030, China; 3 P.O. Box 291, 138 Yi Xue Yuan Road, Shanghai, 200032, China

**Keywords:** Electroacupuncture analgesia, Orexin A, Post-laparotomy pain, OX1R, SB-334867

## Abstract

**Background:**

Orexin A (OXA, hypocretin/hcrt 1) is a newly discovered potential analgesic substance. However, whether OXA is involved in acupuncture analgesia remains unknown. The present study was designed to investigate the involvement of spinal OXA in electroacupuncture (EA) analgesia.

**Methods:**

A modified rat model of post-laparotomy pain was adopted and evaluated. Von Frey filaments were used to measure mechanical allodynia of the hind paw and abdomen. EA at 2/15 Hz or 2/100 Hz was performed once on the bilateral ST36 and SP6 for 30 min perioperatively. SB-334867, a selective orexin 1 receptor (OX1R) antagonist with a higher affinity for OXA than OXB, was intrathecally injected to observe its effect on EA analgesia.

**Results:**

OXA at 0.3 nmol and EA at 2/15 Hz produced respective analgesic effects on the model (P<0.05). Pre-surgical intrathecal administered of SB-334867 30 nmol antagonized OXA analgesia and attenuated the analgesic effect of EA (P<0.05). However, SB-334867 did not block fentanyl-induced analgesia (P>0.05). In addition, naloxone, a selective opioid receptor antagonist, failed to antagonize OXA-induced analgesia (P>0.05).

**Conclusions:**

The results of the present study indicate the involvement of OXA in EA analgesia via OX1R in an opioid-independent way.

## Background

Acupuncture has been used in China since ancient times to relieve many different types of pain. This approach has few side effects and is considered to be a new, effective alternative medicine in Western countries. However, the mechanism underlying acupuncture analgesia remains unclear. Although the opioid system is definitely involved in acupuncture analgesia, naloxone, an opioid receptor antagonist, can only partially block the analgesic effect of acupuncture [1-4]. Also, in some studies, naloxone failed to reverse pain thresholds that had been elevated by acupuncture [5,6]. Studies show that acupuncture can regulate many types of neuropeptides besides opioids, i.e. orphanin FQ, cholecystokinin, neuropeptide Y [7,8], neurokinin A, and substance P [8,9], to achieve therapeutic as well as analgesic effects [10-12]. The above studies showed that acupuncture analgesia involves some other opioid independent pathway, and may require the participation of neuropeptides.

Discovered in 1998, orexin (also known as hypocretin/hcrt) encodes a hypothalamic neuropeptide precursor protein that gives rise to two mature neuropeptides, orexin A (OXA) and orexin B (OXB), by proteolytic processing. Hcrt mRNA is mainly expressed in the lateral and perifornical areas of the posterior hypothalamus [13,14]. OXA and OXB bind to the orphan G-protein coupled receptors, orexin-1 receptor (OX1R or HCRTR1), which is highly selective for OXA, and orexin-2 receptor (OX2R or HCRTR2), which is nonselective for OXA and OXB [14]. This neuropeptide arrangement functions in multiple physiological processes, such as the regulation of sleep and arousal, feeding behavior, metabolism and nociception. Growing evidence from morphological and behavioral studies has demonstrated the anti-nociceptive role of orexins, particularly OXA. [15-18]. Central nervous system (CNS) administered (intracranial ventricle or intrathecal injection) OXA can suppress mechanical allodynia and thermal hypersensitivity in multiple pain models [19-22]. In a rat model of post-operative pain, the antinociceptive effects of OXA were more remarkable than those of OXB, and the effects of OXA were completely blocked by adenosine type 1 receptor antagonists [23]. Purinergic P(2X) and glycine receptors are proposed to be involved in orexin-induced spinal antinociception. Endocannabinoids and nociceptin/orphanin FQ were found to interplay with orexins in nociceptive processing [24]. The antinociceptive effects of intracranial ventricle injection (i.c.v.) of OXA were greater in histamine H1 receptor or H2 receptor knockout mice than in wild-type mice with pain conditions [25]. In addition, orexinergic projections were identified in periaqueductal gray matter (PAG), the rostral ventral medulla, the dorsal horn and dorsal root ganglion [26-28]. The anti-nociceptive effect of orexins could not be blocked by naloxone at a dose that blocked dynorphin-induced analgesia [29]. These studies suggest OXA may be a potential analgesic substance that acts in an opioid independent manner.

To date, there are few reports on the function of OXA in acupuncture analgesia. Whether CNS OXA levels change after surgery or if OXA is involved in electroacupuncture (EA) analgesia remains unknown. The present study was designed to monitor OXA changes post-surgery and its role in EA analgesia. Using a modified post-laparotomy pain model, which is more closely related to clinical use, the present study was intended to (1) examine the change in OXA levels both in the rat model of post-laparotomy pain and after EA treatment of the model; (2) evaluate the modified rat model of post-laparotomy pain and observe the analgesic effect of intrathecal injected OXA and EA application on the model; (3) test the inhibitory effect of the selective OX1R antagonist, SB-334867, on OXA and EA analgesia; and (4) further verify whether OXA analgesia was independent of the opioid system in the rat model of post-laparotomy pain.

## Methods

A rat model of post-operative pain [30] was adopted and modified. Fentanyl, an opioid μ-receptor agonist and a clinically used analgesic, was selected to evaluate pain state post-laparotomy. Acupuncture and OXA were separately employed in the pain model to test whether they had an analgesic effect. A selective OX1R antagonist, SB-334867, was intrathecally injected to test the possible involvement of OXA in acupuncture-induced analgesia. Naloxone was applied before OXA injection to investigate whether it could block OXA analgesia.

### Animals

Adult male Sprague–Dawley rats (200–220 g) were purchased from the BK Company (Shanghai, China) and allowed to acclimate for one week prior to the beginning of experiments. To minimize possible disturbances, the animals were housed in a quiet room close to the operating room with a 12:12-h light/dark cycle. Room temperature was maintained at 20 ± 2°C. Food pellets and water were supplied *ad libitum*. All protocols for this study were approved by the Animal Care and Use Committee from Shanghai Medical College, Fudan University, and the experiments were conducted in accordance with IASP guidelines for pain research in animals [31].

### Animal husbandry

Animals tend to explore new environments. This may affect their sensitivity to external stimuli [32]. To minimize interference and avoid stress-induced analgesia from the behavioral tests, 4 days prior to the operation, the animals were each placed out of sight of one another in the behavioral testing environment, which was an elevated separated chamber (21 cm × 27 cm × 15 cm) with a mesh floor. The duration of exposure to this area was successively increased in the next 4 days (1 h for the 1st day, and 2, 4, 6 h each for the remaining 3 days). On the day before the operation, when rats were placed on the mesh floor, they immediately sat still or began grooming, indicating that they had adjusted to the environment.

### Surgery

A rat model of laparotomy pain was established according to a previous report [30] and was modified to meet the aims of the present study. Anesthesia was maintained by diethyl ether (5% (v/v)) via a nose cone. The deepness of anesthesia was measured as follows: 1) the righting reflex disappeared (the rat could lie on its back); 2) muscular strength class 0 (the limbs of the rat fell down when lifted and dropped by the surgeon); 3) muscular tension disappeared (the limbs of rat could be stretched by the surgeon freely). When the deep anesthesia was achieved, diethyl ether inhalation was ceased for several minutes and then restarted. Signs of nociception would appear if the inhalation ceased for a relatively long time or the diethyl ether was exhausted. Such signs are muscular contraction, tail flick, righting reflex and muscular tension. After the preliminary experiments, we were familiar with the anesthesia process and able to perform the experiment well.The abdomen was shaved and sterilized with 75% (v/v) ethyl alcohol. A midline incision (changed from 3 cm to 6 cm long) was made on the abdominal wall, and the viscera were gently manipulated with the surgeon’s index finger for 2 min. Next, the abdominal wall was closed with 4–0 sutures. The procedure was completed within 10 min. Animals in the sham model group were operated as per the model group, except for the abdominal incision. Only male Sprague–Dawley rats were selected in the experiments to avoid possible gender effects on pain thresholds [33].

### EA application

For animals in the groups that received EA stimulation, EA was applied via two stainless steel needles (0.3 mm in diameter and 25 mm long) connected with the output terminals of an EA apparatus, the HANS Acupoint Nerve Stimulator (LH202H, Beijing, China). The needles were vertically inserted into bilateral ST36 *(Zusanli)* points to a depth of 5 mm and SP6 *(Sanyinjiao)* points to a depth of 3 mm. The stimulator was turned on, and the frequency was adjusted to 2/15 Hz or 2/100 Hz, while the current was adjusted to 1–3 mA. At this current, slight shaking of the legs was induced. The time course of EA application was 30 min, and the operation was conducted at 10 min for approximately 10 min. For rats in the sham EA group, needles were inserted into the bilateral ST36 and SP6 points with no current stimulation.

### Enzyme-linked immunosorbent assay (ELISA)

#### Tissue processing for ELISA

Rats in the four groups (normal, model, EA 2/15 Hz and EA 2/100 Hz) were sacrificed 1 h after surgery or EA. Samples were collected from the hypothalamus, PAG and spinal cord (T13-L5). Brain regions were dissected according to conventional definitions [34]. Peptide extraction was performed according to a previously described method [35], with slight modifications. Each sample block was put into a microtube and weighed and then immersed in 0.5 M acetic acid at a ratio of 1:10 and boiled for 10 min. Samples were homogenized at 40,000 rpm for 15 s and this process was repeated three times, each with 15 s interval. Samples were then centrifuged for 20 min at 5500 rpm. The supernatants were air dried under a hood overnight, and the dried samples were subsequently stored at −80°C.

#### ELISA quantification of orexin A

Dried samples stored at −80°C were used for OXA measurements, which were quantified using the standard ELISA kit (EK-003-30) obtained from Phoenix Pharmaceuticals Inc. (Belmont, CA, USA) according to the manufacturer instructions. ELISA was chosen to minimize the usage of radioactive materials and, both ELISA and RIA methods used to quantify orexin have been evaluated and used [36,37]. Optical densities of the 96-well microplates were read using an assay reader (Thermal Spectronic, Madison, WI, USA). After averaging the results of duplicate wells, the peptide value of each sample was calculated as pg/mg wet tissue.

### Drug injections

Fentanyl citrate, an opioid μ-receptor agonist and a clinically used analgesic, and the control (normal saline 0.2 mL) were intramuscularly (i.m.) injected. SB-334867 (Tocris, UK, dissolved in dimethyl sulfoxide [DMSO]), naloxone (Sigma, MO, USA, dissolved in normal saline) or OXA (Tocris, UK, dissolved in normal saline) were intrathecally injected. Identical volumes of drug vehicle were administered for comparison with drug-treated groups in each experiment.

For intrathecal injections, a modified method of the direct transcutaneous intrathecal injection was used [38]. After being anesthetized, rats were shaved from the lower back and sterilized. A cylindrical glass bottle (60 mm in diameter, high-temperature sterilized) was put under the rat’s abdomen so that its lower back was curved. The surgeon’s thumb and middle finger held the bilateral posterior superior iliac spine, and the index finger palpated the highest spinous process to locate the sixth lumbar (L6) spinous process. A 10-μL Microliter Syringe was inserted from the caudal end, immediately lateral to the L6 spinous process at a 45° angle to the vertebral column and was pushed slowly toward the cranioventral direction. When a sudden lateral tail movement occurred after penetration of the ligamentum flavum (felt as a pop), the drug or vehicle was injected slowly for 30 s. The syringe was held for 10 s more before removal to prevent the outflow of the drug. In a preliminary study we confirmed the accuracy of intrathecal injection to L6 with 2% (w/v) Evans blue. One hour after injection, Evans blue can be seen throughout the spinal cord, as well as the superior cerebellar pool and the midbrain aqueduct, but was difficult to detect in the lateral ventricle and third ventricle.

### Behavioral testing

The von Frey filaments (Stoelting Inc., USA) were used to identify the severity and duration of mechanical allodynia state in rats according to previously published literature [39,40]. We introduced the abdominal constriction threshold (ACT) in addition to the paw withdrawal threshold (PWT) to select better assessments for this model.

ACT and PWT were tested consecutively. Filaments were applied from underneath the chamber through openings in the mesh floor to an area 5 mm adjacent to the abdominal wound line (ACT) or to an area in the plantar surface of the left foot (PWT). The filaments produced an upward force to induce abdominal constriction or paw withdrawal. A preliminary experiment was conducted to select the proper sequential filaments, as shown in Table 1.

**Table 1:** Selection of sequential filaments for ACT and PWT before and after operation

	**Before operation**	**After operation**
*ACT*	8.0 g, 10.0 g, 15.0 g, 26.0 g	0.16 g, 0.4 g, 0.6 g, 1.0 g, 2.0 g, 4.0 g, 6.0 g, 8.0 g, 10.0 g, 15.0 g, 26.0 g
*PWT*	6.0 g, 8.0 g, 10.0 g, 15.0 g, 26.0 g	4.0 g, 6.0 g, 8.0 g, 10.0 g, 15.0 g, 26.0 g

Each of the sequential von Frey filaments was applied once for 3 s starting with the minimum and continuing until an abdominal constriction or foot withdrawal response occurred. After a 3-min test-free period, each filament was again applied once, beginning with the minimum, until a response was elicited. This was repeated a third time 3 min later. The lowest force from the three tests producing a response was considered the ACT or PWT. The cutoff value, 26.0g, was recorded even if there was no response to this force. The animals were tested at baseline, 0.5, 1, 2, 4, 6 and 24 h after surgery. Time 0 was defined as the end of the surgery or EA application.

The treatment regimes were:

(a) No drug (or EA) treatment:

normal group, model group (rats undergone surgery) and sham model group (anesthesia only rats) (n=8 per group).

Post-operative treatment with fentanyl:

To fully evaluate the pain state of the laparotomy model, rats received a fentanyl injection (5, 10, 20 μg/kg, i.m., n=8 per group) or drug-vehicle (saline 0.2 mL, n=8) immediately after surgery at time 0.

(b) Post-operative treatment with OXA:

To verify whether OXA has an analgesic effect on the model, rats received intrathecally administered OXA (0.1, 0.3 and 1.0 nmol, n=8 per group) or drug-vehicle (normal saline 10 μL, n=6) after surgery at time 0.

Perioperative treatment with EA:

To explore the EA analgesic effect on the rat model, EA was applied perioperatively for 30 min, i.e., EA stimulation (2/15 Hz or 2/100 Hz) begun 10 min before surgery and ended 10 min after surgery. During the middle 10 min of EA the surgery was completed. Thus, when EA was applied in the experiment, time 0 was defined as the end of EA.

(c) Pre-operative treatment with SB-334867on OXA analgesia:

To explore whether OXA produces its analgesic effect via OX1R, the selective OX1R antagonist SB-334867 (30 nmol, n=7) or drug-vehicle (DMSO, 10 μL, n=8) was intrathecally administered prior to surgery OXA with the minimum effective dose selected from (c) was intrathecally administered post-surgery.

Pre- or post-EA treatment with SB-334867:

To examine whether OXA participates in EA analgesia via OX1R, the selective OX1R antagonist SB-334867 (30 nmol, n=7-8 per group) or drug vehicle (DMSO 10 μL) was intrathecally administered prior to or post-EA-treatment.

(d) Pre-EA treatment with naloxone

To explore whether OXA analgesia was independent of the opioid system, the opioid receptor antagonist naloxone 28 nmol or drug vehicle (saline 10 μl) was intrathecally injected before surgery OXA, was intrathecally injected after surgery.

### Statistical analysis

Statistical analyses were performed using SPSS 13.0 software. Threshold response data were represented as the mean ± standard error of means (SEM). Between groups, ACT and PWT data at each time point were analyzed by a one-way analysis of variance (ANOVA) followed by a post hoc least significance difference test (LSD). A probability (p) value of < 0.05 was considered to be statistically significant.

## Results

### Changes to OXA levels in the hypothalamus, PAG and spinal cord following laparotomy and/or EA treatment

**Figure 1:**
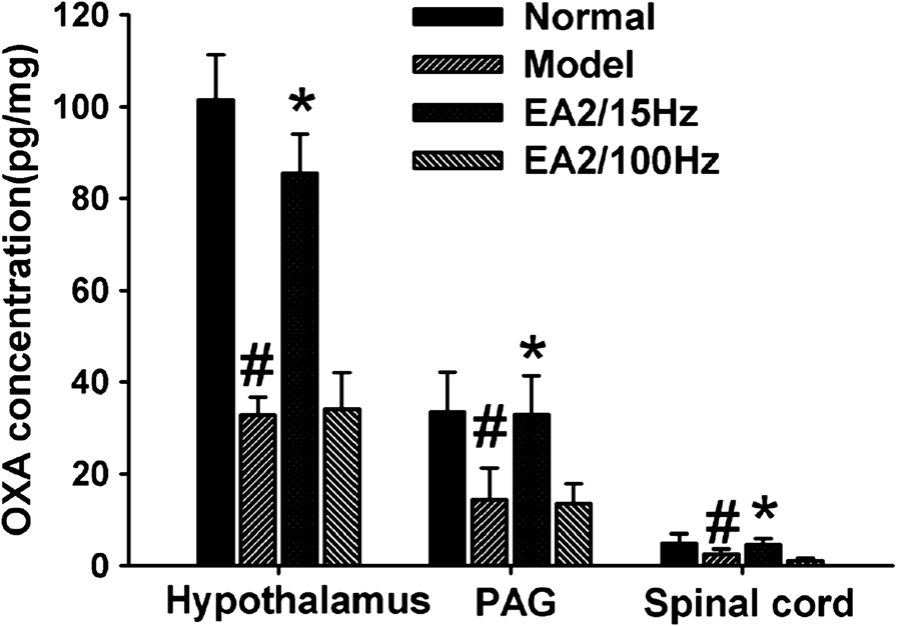
**OXA levels determined by ELISA in the rat hypothalamus, PAG and spinal cord in the normal, model, EA 2/15 Hz and EA 2/100 Hz groups.** Data are presented as the mean ± SEM (n=4 in each group). #P<0.05 versus normal group, *P<0.05 versus model group. PAG: periaqueductal gray matter.

The ELISA tests showed that there was a significant decrease of OXA peptide levels in the hypothalamus, PAG and spinal cord in rats after surgery (P<0.05) when compared with the normal group. EA 2/15 Hz reversed the decrease in OXA levels in the above regions in model rats (P<0.05), whereas the EA2/100 Hz group showed no change (P>0.05; Figure 1).

### Induction of mechanical allodynia in the rat model of post-laparotomy pain

In behavioral tests, no statistical difference was found in the baseline thresholds measured by PWT or ACT among the tested groups before surgery (P>0.05 by ANOVA).

**Figure 2:**
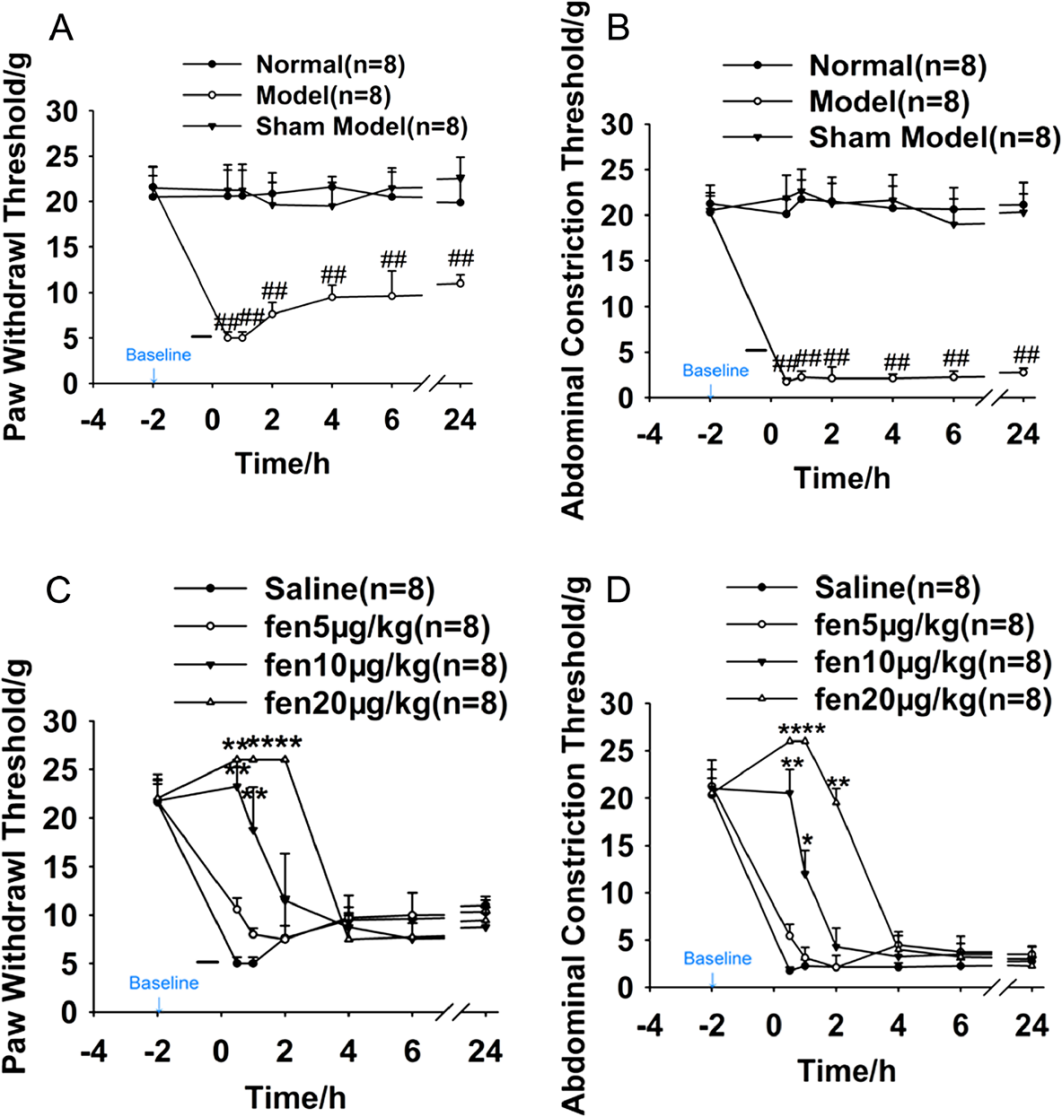
**Mechanical threshold changes (in grams) after surgery with or without intramuscular injection of fentanyl are presented as the mean ± SEM at the indicated time points post-surgery. A**, **B**: abdominal and paw mechanical changes among normal, sham model and model groups after surgery. **C**, **D**: abdominal and paw mechanical changes among saline, fentanyl 5, 10 and 20 μg/kg groups. ##P<0.01 versus the sham model group, #P<0.05 versus the sham model group; *P<0.05, **P<0.01 versus the saline group. Short black lines represent the surgery duration. Fen: fentanyl, BL: baseline.

No significant difference in mechanical thresholds was detected between the normal and sham model group (P>0.05), as shown in Figure 2A and B. Animals in the model group displayed significant allodynia to mechanical stimulation in both the hind paw and the abdomen (P<0.05). PWT significantly decreased at 0.5 h post-surgery in the model group when compared with the sham model group (P<0.01). ACT also decreased in the model group when compared with the sham model group (P<0.01). These data provide behavioral evidence for the postoperative pain model.

### Relief of mechanical allodynia with fentanyl in the rat model of post-laparotomy pain

The μ-opioid receptor agonist fentanyl was intramuscularly (i.m.) injected immediately after surgery at 5, 10 and 20 μg/kg. Figure 2C and D show that 10 and 20 μg/kg of fentanyl dose-dependently increased PWT and ACT (P<0.05 compared with the saline group). In the following experiment, ACT was chosen as a mechanical threshold parameter because it represented the local allodynia state and was relatively stable compared with PWT. These data independently provide evidence that laparotomy pain can be relieved by analgesia in this model.

### Effect of postoperative OXA injection in the rat model of post-laparotomy pain

**Figure 3:**
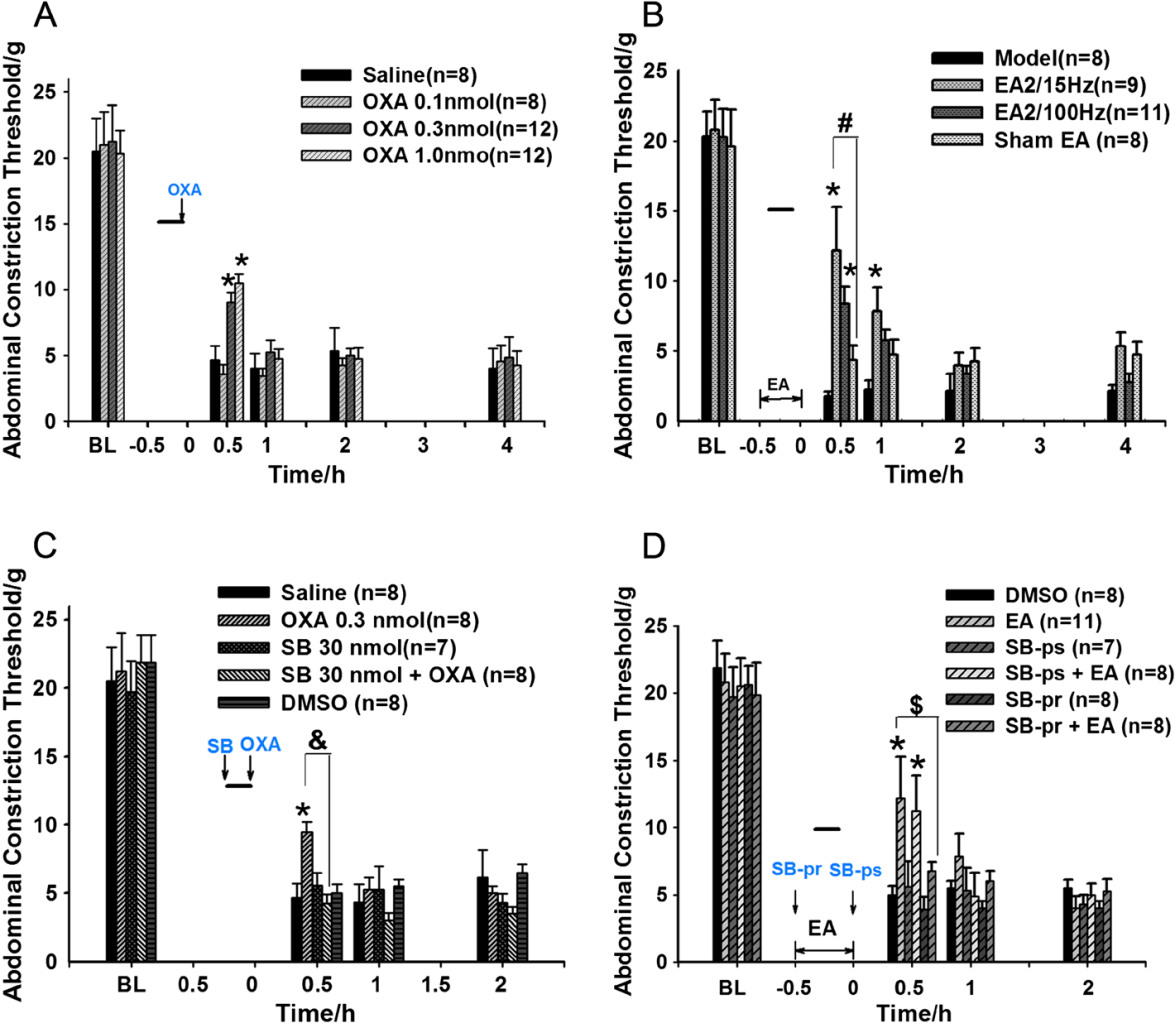
**Effect of post-surgical OXA injection (A) and perioperative EA (B) on the rat model of post-laparotomy pain, and effect of SB-334867 on OXA analgesia (C) and EA analgesia (D).** Data are presented as the mean ± SEM at the indicated time points post-surgery. *P<0.05 versus the saline/model/DMSO group. #P<0.05 versus the sham EA group, &P<0.05 versus the SB-334867 + OXA group, $P<0.05 versus the EA group. Short black line represents the surgery duration. The double-headed arrow represents the EA duration. BL: baseline. SB-pr: pre-surgical injection of SB-334867; SB-ps: post-surgical injection of SB-334867.

OXA at a concentration of 0.1, 0.3 and 1.0 nmol or saline was intrathecally injected into animals immediately after surgery. Figure 3A shows that 0.1 nmol OXA did not have an analgesic effect after surgery, whereas OXA at 0.3 and 1.0 nmol increased ACT significantly at 0.5 h compared with the saline group (P<0.05). There was no statistically significant difference in ACT between the OXA 0.3 nmol and OXA 1.0 nmol groups. Thus, the 0.3 nmol intrathecally-injected dose was chosen in all subsequent experiments.

### Effect of perioperative EA application in the rat model of post-laparotomy pain

Perioperative EA stimulation was applied bilaterally to the ST36 and SP6 acupoints at 2/15 Hz or 2/100 Hz for 30 min. Surgery was performed during the middle 10 min of EA stimulation. Figure 3B shows that EA at 2/15 Hz significantly increased ACT in the 1st h after surgery compared with the model group (P<0.05). At 0.5 h, the EA 2/15 Hz group also showed a significantly difference in ACT when compared with the sham EA group (P<0.05). EA at 2/100 Hz produced significant analgesia as measured by ACT compared with the model group within 0.5 h (P<0.05). Therefore, EA stimulation using 2/15 Hz was used for all subsequent experiments.

### Effect of preoperative intrathecal injection of SB-334867 on OXA analgesia in the rat model of post-laparotomy pain

The OX1R antagonist SB-334867 (30 nmol) was intrathecally injected prior to surgery. Figure 3C shows that there was no significant difference in ACT among the SB-334867 group, DMSO group and saline group at 0.5 h post-surgery (P>0.05). The SB-334867 30 nmol + OXA group showed a significant difference when compared with the OXA 0.3 nmol group (P<0.05).

### Effect of pre-surgery intrathecal injection of SB-334867 on EA analgesia in the rat model of post-laparotomy pain

SB-334867 was intrathecally injected into the rat model in addition to EA application. SB-334867 (30 nmol) had no obvious effect on the ACT at 0.5 h when compared with the DMSO group (P>0.05 by one way ANOVA with LSD test). Pre-surgical treatment with SB-334867 (SB-pr) significantly decreased EA analgesia (P<0.05 by one way ANOVA with LSD test) (Figure 3D), while post-surgical administration of SB-334867 (SB-ps) did not decrease the EA analgesic effect.

### Effect of pre-treatment with naloxone on OXA analgesia and pre-treatment with SB-334867 on fentanyl-induced analgesia

**Figure 4:**
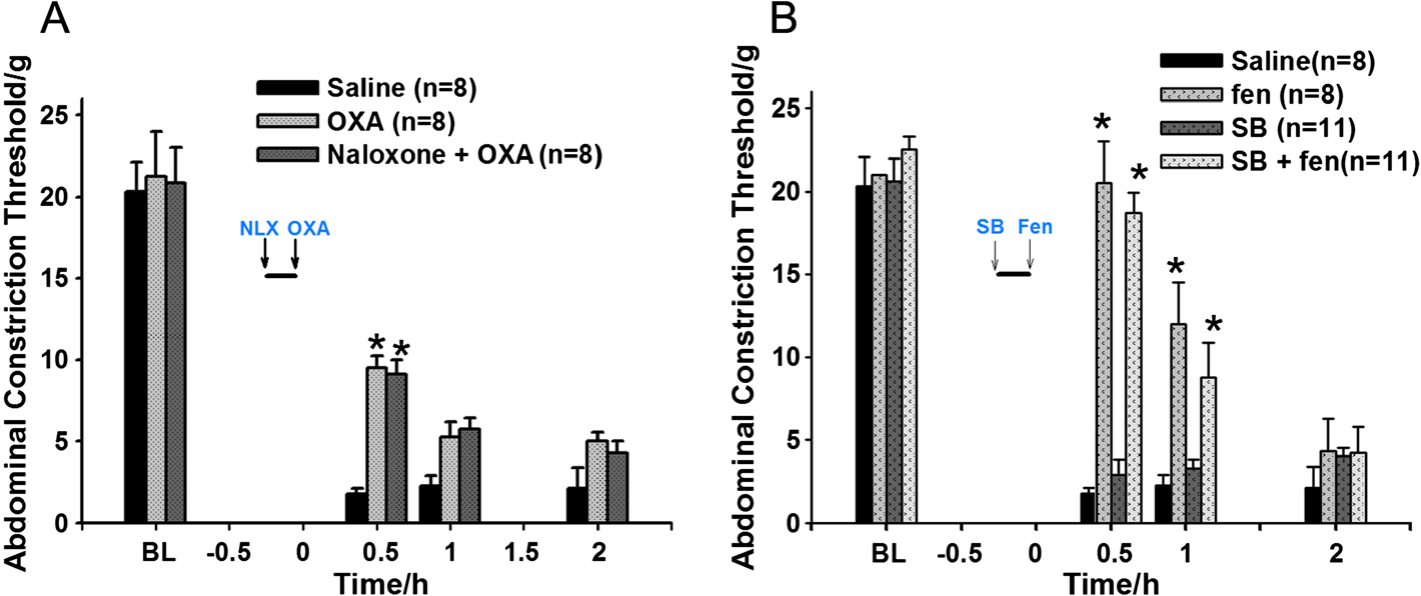
**The OXA analgesic effect was not blocked by intrathecal administration of naloxone (A).** The fentanyl-induced analgesic effect was not blocked by intrathecal administration of SB-334867 (**B**). Data are presented as the mean ± SEM. *P<0.05 versus saline group. Short black line represents the surgery duration. BL: baseline; NLX: naloxone; Fen: fentanyl; SB: SB-334867.

Naloxone was intrathecally injected at a dose of 28 nmol pre-surgery followed by an intrathecal injection of OXA (0.3 nmol) post-surgery. At 0.5 h post-OXA injection, the OXA 0.3 nmol group and the OXA + Naloxone group both showed a significant difference in the ACT when compared with the saline group (P<0.05), whereas there was no difference between the OXA group and the OXA + Naloxone group (P>0.05) (Figure 4A).

In another experiment, we intrathecally injected SB-334867 pre-surgery followed by an intramuscular injection of fentanyl post-surgery. Results showed there was no significant difference between the SB-334867 group and model group, or between the SB-334867 + fentanyl group and fentanyl group (P>0.05; Figure 4B).

## Discussion

The present study showed that after laparotomy, OXA levels in the hypothalamus, PAG and spinal cord decreased significantly. Bilateral EA at ST36 and SP6 elevated OXA levels in the above regions. Rats displayed a local and systematic mechanical allodynia state after laparotomy. The allodynia could be relieved by fentanyl in a dose-dependent manner with a limited time course of 2 h. OXA at 0.3 nmol and EA at 2/15 Hz both showed analgesic effects in the rat model. SB-334867 30 nmol blocked OXA-induced analgesia. Pre-surgical, but not post-surgical, administration of SB-334867 treatment significantly reduced EA analgesia. These results suggested the involvement of OXA in acupuncture analgesia via OX1R. As we focused on laparotomy induced pain, indexes such as blood pressure, heart rate and breath during surgery were not included in the present study.

### Relevance and validity of the model and pain assessment

In this study, we used the mechanical measurement of ACT in addition to PWT to evaluate the pain state of the animal. PWT decreased significantly after laparotomy; this effect lasted for over 24 h, which is in agreement with previous reports [41,42]. Because paw withdrawal is a complex reflex that requires spinal and supraspinal reflexes and includes cerebral involvement, this reflex can be used to represent the systemic pain state. The hind paw test is commonly used to evaluate mechanical pain, including hind paw pain, patellar osteoarthritis, abdominal incisional pain and tibial cancer pain, among others. In abdominal pain following surgery, nerves innervating the hind paw (superficial and deep branches of the peroneal nerve as well as medial and lateral plantar nerves) terminate in the lumbar-sacral segments L6–S2. The surgical site is served by the lumbar spinal cord segments L1–L4, with some input from T13. Assessments of the pain state of the abdomen, not the hind paw, could be more consistent with typical clinical scenarios. The idea of using the ACT came from the “abdominal constriction score” or “abdominal withdrawal response” when assessing visceral inflammatory pain. We introduced the ACT to evaluate abdominal mechanical allodynia and compared it with PWT. As shown in Figure 1A and B, the same tendency of ACT and PWT was observed in the model group and fentanyl groups, but ACT remained at a relatively steady level, whereas PWT gradually increased towards baseline levels. Thus, the ACT could be more suitable than other tests in assessing abdominal pain.

### Acupoints and EA frequency selection

In the present study, we selected the bilateral acupoints of ST36 and SP6 to relieve laparotomy pain. In Chinese medicine, pain is believed to be caused by either Qi flow stagnation or Qi-blood insufficiency. In the laparotomy case, abdominal pain is caused by incision, which induces Qi and blood stagnation. ST36 and ST6 have been reported to alleviate many kinds of abdominal pain by regulating and activating Qi and blood movements. When Qi flows smoothly, the pain is relieved. Some studies have found that after EA at ST36, high IL-1 Receptor-I mRNA expression of PAG due to peripheral inflammation was inhibited and the high pain threshold significantly increased [43]. This indicates the analgesia of EA at ST36 may be a consolidation of signals at the PAG level. In the present study, we have found that EA at the bilateral ST36 and SP6 increased the mechanical threshold of rats after laparotomy, as compared with the model group.

EA analgesia is achieved by integration of the wave, pulse width, frequency and intensity of stimulation on acupoints. Among these factors, frequency is a key factor affecting the analgesic effect of EA. We selected 2/15 Hz and 2/100 Hz for EA administration and compared the analgesic effects of these two frequencies. Our results show that 2/15 Hz produced a better analgesic effect in the rat model. Experimental studies have shown that different frequencies can cause the brain and spinal cord to release different types of opioid peptides, which may have different effects [44]. EA stimulation at 2 Hz accelerates the release of endorphins in the brain and large amounts of enkephalin in the spinal cord [45-47], while 15 Hz EA stimulation causes the release of endorphins, beta-endorphin, endomorphin and dynorphin, and 100 Hz EA stimulation causes the spinal cord to release dynorphin [10,48-51]. Thus, 2/15 Hz EA stimulation may accelerate the release of many different endogenous opioid peptides [52], each of which strengthens the effect of the others. Therefore, this frequency of EA can produce a strong analgesic effect.

### OXA and EA analgesia

Recent studies show that orexins, especially OXA, may play an important role in pain modulation. Previous studies showed that OXA injection into the posterior hypothalamus of rats reduced the afference of facial A- and C-fibers to electrical and heat stimulation, while OXB increased the afferent response to heat stimuli [53]. In addition, prepro-orexin (PPO) knockout mice displayed significant hyperalgesia and reduced stress-induced analgesia compared with wild-type mice, suggesting that pain and stress stimulate the orexin system to modulate the pain transmission process [54]. Orexin participates in the nociceptive process both at the spinal cord and supraspinal cord level and may also be involved in the descending inhibitory system of pain regulation [26-28].

The present study showed that intrathecal injection of OXA can relieve the pain state induced by laparotomy, and that this effect was antagonized by the selective OX1R antagonist SB-334867. The OXA analgesia effect of 0.3 nmol and 1.0 nmol was not dose-dependent. Similar observations were made by Bingham et al. [21]. This result may be because OX1R was already saturated for OXA at the dose of 0.3 nmol. In the present study, pre-surgery treatment with SB-334867, instead of post-surgical SB-334867, blocked EA analgesia. We can infer it is because EA activated OXA release to achieve the analgesic effect, which was proven by the ELISA. OXA has a much higher affinity for OX1R than OXB. The above results strongly indicate the involvement of orexin, particularly OXA in EA analgesia. However we can’t completely exclude the possibility that OXB is also involved. In another experiment, the analgesic efficacy of intrathecal injection of OXA was superior to OXB in the paw incision induced pain model [23]. Nevertheless we can infer from the present results that OXA is a more potent factor in participating in EA analgesia than OXB.

Because EA mainly achieves its analgesic effect by stimulating the release of opioid peptides, whether SB-334867 blocked EA in an opioid-dependent way was also studied. We conducted experiments using intrathecal injections of naloxone, which has an extremely high affinity for μ-opioid receptors and a lower affinity at κ- and δ-opioid receptors in the CNS. Therefore, naloxone mainly antagonizes the analgesic effect of endorphins, as well as part of the effects of dynorphin and enkephalin. There is strong evidence that naloxone can block EA analgesia; however, inhibition is not complete. Therefore, we can’t exclude the role of opioids in acupuncture analgesia. Unfortunately, we did not repeat the naloxone + EA experiment because our study focused on OXA involvement in acupuncture analgesia.

We found that naloxone (28 nmol), at a dose that blocked dynorphin-induced analgesia, was unable to block OXA-induced analgesia. This result is in accordance with previous studies [21,29]. Also, the OX1R receptor antagonist SB-334867, at a dose that blocked EA analgesia, did not block fentanyl-induced analgesia.

In addition, OXA levels in the hypothalamus, PAG and spinal cord also decreased significantly after laparotomy and were reversed by EA. The above areas are included in the descending inhibitory system. This result has shown that EA can regulate the descending inhibitory system to achieve an analgesic effect. We can only infer that the possible reason for an OXA decrease in the model group is that the laparotomy is a traumatic injury to rats and induces a stress response, which causes exhaustion of OXA in the CNS. OXA is produced through hydrolysis of its precursor PPO by the proteolytic enzyme during the process of axonal transport [55]. The mechanism why EA increased OXA levels remains to be explored. One possible mechanism is that EA stimulation promotes the axon transmission of OXA and accelerates proteolysis, which in turn increases OXA production and release [56]. Whether EA initiates the rapid response of cells to achieve the subsequent effect remains to be clarified. In the present study, only 2/15 Hz increased OXA levels in the above areas. This may provide further evidence that different frequencies of EA stimulate the release of different peptides.

## Conclusion

Although orexin A (OXA) is a newly discovered potential analgesic substance, whether OXA is involved in acupuncture analgesia remains unknown. In this study we have shown that EA reversed the OXA decrease in rats post laparotomy. Intrathecal administered of OX1R antagonist SB-334867 antagonized OXA analgesia and attenuated the analgesic effect of EA, but did not block fentanyl-induced analgesia. In addition, naloxone failed to antagonize OXA-induced analgesia. The results of the present study indicate the involvement of OXA in EA analgesia via OX1R in an opioid-independent way.

## Competing interests

The authors declare that there are no conflicts of interest to declare.

## Authors’ contributions

XMF WLM and FX carried out the study design, experimental work, data collection and interpretation, literature review, and manuscript preparation. YQW and QLMY provided assistance with applying for the supporting grant. JWJ provided an excellent research environment and participated in discussion and coordination. GCW supervised the work, evaluated the data, and corrected the manuscript for publication. All authors read and approved the final manuscript.

## Pre-publication history

The pre-publication history for this paper can be accessed here:

http://www.biomedcentral.com/1472-6882/12/225/prepub
